# A Retrospective Study of Cryptorchidectomy in Horses: Diagnosis, Treatment, Outcome and Complications in 70 Cases

**DOI:** 10.3390/ani10122446

**Published:** 2020-12-21

**Authors:** Paola Straticò, Vincenzo Varasano, Giulia Guerri, Gianluca Celani, Adriana Palozzo, Lucio Petrizzi

**Affiliations:** Faculty of Veterinary Medicine, University of Teramo, Località Piano D’Accio, 64100 Teramo, Italy; pstratico@unite.it (P.S.); vvarasano@unite.it (V.V.); gcelani@unite.it (G.C.); apalozzo@unite.it (A.P.); lpetrizzi@unite.it (L.P.)

**Keywords:** cryptorchidism, laparoscopy, orchiectomy, horse

## Abstract

**Simple Summary:**

Cryptorchidism is the failure of one or both testes to descend into the scrotum and is considered to be one of the most common developmental disorders in horses. The aim of the study was to review medical records of horses referred for uni- or bilateral cryptorchidism. It was observed that the Western Riding horse breeds were the most affected, and that left abdominal and right inguinal retentions were the most frequent. Transabdominal ultrasound was the most reliable diagnostic tool to localize the retained testis. Standing laparoscopic and open inguinal cryptorchidectomy were elected as the surgical treatment of choice, in case of abdominal retention and inguinal retention respectively. For incomplete abdominal retention, laparoscopy was the preferred treatment, even though an open inguinal approach was a viable option for the concurrent removal of the descended testis.

**Abstract:**

The aim of the study was to investigate the breed predisposition and the diagnostic and surgical management of horses referred for cryptorchidism. The breed, localization of retained testis, diagnosis, type of surgical treatment and complications were analyzed. Seventy horses were included in the study; the Western Riding horse breeds were the most affected (Quarter Horse 34/70, 48.5%; Appaloosa 9/70, 12.8%). In unilateral cryptorchids (65/70, 92.8%) the most common location for a retained testis was the left abdomen (28/65, 43%), while in bilateral cryptorchids (5/70, 7.1%), bilateral abdominal retention was the most frequent (3/5, 6%). Information about testis localization was achieved through transabdominal ultrasound (30/49 cases, 61.2%), through *per rectum* palpation (21/49 cases, 42.9%) and through inguinal palpation (14/49 cases, 28.9%). Cryptorchidectomy was achieved with standing laparoscopy (44/70 cases, 62.8%), or with open inguinal orchiectomy in general anesthesia (26/70 cases, 37.2%). Complications during laparoscopy were spleen puncture (1/44, 2.2%), a self-limiting bleeding from the spermatic cord (10/44 cases, 22.7%), hyperthermia (3/44 cases, 6.8%), and emphysema (15/44, 34%). During inguinal open cryptorchidectomy difficulties with identifying the inguinal testis during surgery (8/26 cases, 30.8%) and a moderate and self-limiting swelling of the inguinal region after surgery (17/26, 65.4%) were observed. For orchiectomy, a standing laparoscopy was confirmed as the preferred procedure for an abdominally retained testis with almost no complications.

## 1. Introduction

Cryptorchidism is the failure of one or both testes to descend into the scrotum and is considered to be one of the most common developmental disorders in horses [1]. In addition to the hypofertility associated with the condition, cryptorchid horses often have undesirable behavior [2,3,4]. Thus, removal of the retained testis is often required [5,6].

According to previous literature, cryptorchidism has the greatest prevalence in Percherons, followed by American saddle horses and Quarter horses [7]. Unilateral retention is more prevalent than bilateral cases, with higher frequencies of left abdominal retention as compared with right sided cases (75% vs. 42%) and of right sided inguinal retention compared with left sided retention (58% vs. 25%) [8].The reported frequency of bilateral retention ranges from 9% to 14%, with abdominal retention being 2.5 times more common that inguinal retention [8,9].

A diagnosis of cryptorchidism is usually reached combining inguinal, rectal palpation, and ultrasonographic findings. Palpation is easy and not time consuming and has an accuracy of 87.5% when performed by an experienced clinician [8]. However, when performed *per rectum* in small breeds and in young or fractious horses, it can be dangerous [10,11,12,13]. In such cases, due to the risks described for rectal palpation, the inguinal and transabdominal ultrasonographic approaches are preferred to the transrectal one, with a sensitivity of 97.5% when combined [14]. In mature horses, the association of transrectal and inguinal ultrasonography can reach a high sensitivity, with a 100% correlation with surgical diagnosis [10].

Several surgical techniques are available for the removal of a retained testis, depending on its location (inguinal vs. abdominal), as well as temperament, size and age of the horse, in addition to the surgeon’s preference and confidence in the technique. Laparoscopic approaches to the retained testis differ from each other, mainly in relation to patient positioning (standing versus recumbent) [15,16] and hemostasis methods [13,15,16,17,18,19,20,21,22,23,24,25].

Because of the excellent visualization of abdominal viscera, easy localization of the retained gonad, reduced surgical time, reduced complications and faster recovery, standing laparoscopic surgery has gained popularity both as a diagnostic and therapeutic tool for the removal of abdominally retained testes, as well as for ovariectomy in equids [19,20,22,26,27,28,29,30]. Standing laparoscopy is preferred to the recumbent approach in order to avoid the costs and risks of general anesthesia, and the hemodynamic challenges related to concurrent recumbency and pneumoperitoneum [31,32]. The development of innovative technologies to achieve better intrabdominal hemostasis has now almost completely overridden the need for extracorporeal or intrabdominal sutures or Nd:YAG laser photocoagulation, with a reduction of surgical time and eliminating the risk of ligature slippage, the cost of preformed ligatures, the advanced training needed for knot tying and the use of laser equipment [17,33,34,35,36,37].

The purpose of the study is to review the clinical records of equine patients referred for cryptorchidism, adding information about breed prevalence, distribution of retained testicles, preoperative localization of the retained testis, surgical management and complications.

## 2. Materials and Methods

The medical records of equids referred and treated for cryptorchidism at the Veterinary Teaching Hospital (VTH) of the University of Teramo from January 2009 to July 2019 were reviewed. Inclusion criteria were the diagnosis of uni or bilateral cryptorchidism, with the request for removal of the retained testis.

The retrospective analyses of medical records included information about signalment, the result of scrotal, inguinal or *per rectum* palpation, the result of ultrasonographic examination (inguinal, transabdominal or transrectal), the type of retention (inguinal, abdominal, incomplete abdominal; unilateral vs. bilateral), the surgical technique (standing laparoscopic vs. recumbent inguinal open), the total anesthesia or sedation time, the total surgery time and any intraoperative and short-term complications.

All horses underwent a thorough physical examination. They were sedated with xylazine hydrochloride (0.5 mg/kg i.v.) and restrained in stocks. The scrotal and inguinal regions were palpated to assess the evidence of a descended or undescended testicle. Testicles located in the scrotum were considered as normally descended. If the testis was palpated in the subcutis or within the inguinal canal, a diagnosis of inguinal cryptorchidism was made. When no testes could be located, abdominal palpation was performed *per rectum*. During transrectal palpation, the vas deferens of the retained testicle passing through the inguinal ring was looked for. If the vas deferens was appreciated running within the inguinal ring, a diagnosis of abdominal incomplete cryptorchidism was made. If the vas deferens was not palpated running through the inguinal ring, abdominal cryptorchidism was diagnosed [8,38].

The ultrasonographic examination was performed bilaterally at the inguinal region [39] and the caudo-ventral abdomen using a 3.5 MhZ convex probe. Isopropyl alcohol 70% and coupling gel were applied to the skin in order to avoid clipping before scanning [14]. When requested, after removal of feces from the rectum, a transrectal ultrasonography was performed with a rectal linear 7.5 MhZ probe [10].

When abdominal retention was diagnosed, the horse was prepared for uni/bilateral standing laparoscopic cryptorchidectomy [20]. Food, but not water, was withheld for 24 h before the procedure. Horses were sedated with a bolus of detomidine HCl (0.01 mg/kg intravenously) and butorphanol tartrate (0.01–0.015 mg/kg i.v.), followed by a continuous rate infusion (CRI) of detomidine (0.5 µg/kg/min for 15 min, followed by 0.3 µg/kg/min for 15 min and finally 0.15 µg/kg/min until 5–15 min prior to the end of the procedure) through the duration of the procedure [40]. Adjunctive doses of butorphanol (0.005–0.01 mg/kg i.v.) were administered if signs of pain were observed. The duration of sedation was recorded, from the initial bolus of detomidine to the interruption of the infusion.

For laparoscopy, on the side of retention or bilaterally in cases of bilateral abdominal retentions, the abdominal flank was clipped and aseptically prepared for surgery. Skin, subcutaneous tissue, and muscles were then infiltrated with 20 mL of 2% lidocaine at the level of each expected laparoscopic portal. After surgical draping, a 1.5 cm skin incision was made in the paralumbar fossa, dorsal to the crus of the internal abdominal oblique muscle equidistant from the tuber coxae and the last rib [21]. Then a 10 mm blunt trocar cannula was introduced into the skin incision, through the abdominal wall, into the peritoneal cavity. A pneumoperitoneum was created connecting the cannula to a CO_2_ insufflator to reach an intrabdominal pressure of 10 to 15 mmHg. One hand-width dorsal to the insufflation portal, another 1.5 cm skin incision was made, and a second 10 mm cannula was inserted into the peritoneal space with the same modality as the previous one. The blunt trocar was removed to be replaced with a rigid telescope (45°, 10 mm diameter, 57 cm long) connected to a video camera. A third 1.5 cm skin incision was made one hand-width ventral to the first, and another cannula was introduced to be used as a second instrument portal.

The abdominal testis was identified, grasped with a 10 mm pair of Claw laparoscopic forceps introduced in the distal portal, and held in place to allow sealing of the testicular vessels, mesorchium and vas deferens with the Ligasure^TM^ system until the testis was completely freed [17]. Finally, a careful inspection for assessing adequate hemostasis was performed, and the excised testicle was withdrawn from the abdomen through the most ventral portal, accordingly enlarged: to this aim the skin incision was widened sharply and the abdominal wall muscles were digitally split along the fibers. In cases of bilateral abdominal retention, the same procedure was repeated on the other flank. At the end of surgery the abdomen was deflated, the muscular incision was closed with one or two cruciate pattern mattress sutures with a 2 USP multifilament absorbable suture (Polisorb, Covidien Italia S.pA., Milano, IT), and the skin incision was closed with wide surgical stainless steel staples (Visistat, Weck 35W, Teleflex, Morrisville, NC, USA).

In the case of unilateral abdominal retentions with a contralateral descended testis, the eutopic testicle was removed using a standing open scrotal approach with emasculation of the spermatic cord [41].

When an inguinal retention was diagnosed, the patient was prepared for a dorsally recumbent, open inguinal cryptorchidectomy, under general anesthesia. Such horses were premedicated with medetomidine (7 µg/kg i.v.) and general anesthesia was obtained with an injection of ketamine (2.2 mg/kg i.v.) and diazepam (0.02 mg/kg i.v.) and maintained with isoflurane in oxygen-enriched air and CRI of medetomidine (3.5 µg/kg i.v.) Twenty minutes before the end of surgery morphine (0.1 mg/kg i.v.) was administered intramuscularly (i.m.) [42,43,44]. The duration of anesthesia was calculated from the induction of anesthesia to the conclusion of surgery [38]. The duration of sedation was calculated from the first injection to the conclusion of surgery.

As described in the literature [45,46] the surgical procedure consisted of an inguinal approach at the side of retention: after reaching the superficial inguinal ring, the inguinal process was identified, everted and opened to identify and remove the testis. If the retention was unilateral, the contralateral scrotal testicle was removed consecutively using an inguinal approach. All surgical wounds underwent closure in order to achieve primary intention healing [41].

Depending on the surgeon’s preference, as well as the age and temperament of the horse, incomplete abdominally retained testicles were removed through standing laparoscopy or an inguinal open approach in general anesthesia. In cases of a recumbent open inguinal approach in general anesthesia, the inguinal process, once identified, was opened to grasp the tail of the epididymis and then expose the ductus deferens, the proper ligament and finally the testis, which was excised after ligation and division of the cord.

The duration of all surgeries was measured from the skin incision to last suture knot and recorded both for laparoscopic and inguinal cryptorchidectomy.

All horses undergoing orchiectomy with first intention healing received perioperative antibiotics (ampicillin 20 mg/kg i.v. and gentamicin 8 mg/kg i.v.); horses that received a standing open scrotal approach with second intention healing received a three-day course of antibiotics (penicillin-dihydrostreptomycin 10,000 U.I. i.m. every 24 h). All patients were administered flunixin meglumine (1.1 mg/kg i.v. every 24 h for 3 days after surgery).

For each procedure intraoperative and postoperative complications were recorded.

## 3. Results

A total of 70 horses underwent cryptochidectomy during the 10-year study period. The population was composed of Quarter Horses (*n* = 34, 48.6%), Appaloosas (*n* = 10, 14.3%), Arab Thoroughbreds (*n* = 6, 8.5%), Italian Saddle Horses (*n* = 6, 8.5%), Paint Horses (*n* = 5, 7.2%), and Frisian Horses (*n* = 4, 5.7%). The remaining equids were of less represented breeds (English Thoroughbred, Warmblood, Spanish Saddle Horse, Haflinger, Pony) for a total of 5 horses (7.2%). Median age was 3 years (range 2 to 12 years).

The scrotal and deep inguinal palpations were always performed, but the results were not always reported in the clinical records. Information about testicle localization before surgery was available in 65/70 cases. The diagnosis was confirmed by *per rectum* palpation in 21/70 cases (30%), by inguinal palpation in 14/70 cases (20%) and by transabdominal ultrasound in 30/70 cases (42.8%). For the remaining patients (4/70), information about testis localization before surgery could not be retrieved.

The majority of patients included in the study had a unilateral retention (*n* = 65, 92.8%; left *n* = 44, 68%; right *n* = 21, 32%), while bilateral cryptorchidism was diagnosed in only 5 cases (7.1%). In cases of unilateral cryptorchidism, the most common location was the left abdomen (28/65, 43%), followed by right inguinal and left incomplete abdominal retentions (in both cases 9/65, 14%). Left inguinal and right abdominal retention represented 7/65 cases (11%), while right inguinal and left incomplete abdominal retentions represented 5/65 (7%). When bilateral cryptorchidism was diagnosed, the most common location was bilateral abdominal (3/5, 60%), followed by bilateral incomplete abdominal and bilateral inguinal retention (in both cases 1/5, 20%) (Table 1).

All horses underwent surgical castration. In 44/70 cases (62.8%) laparoscopic cryptorchidectomy was performed, while 26/70 cases (37.1%) received an open inguinal orchiectomy. A laparoscopic cryptorchidectomy was chosen in all cases of unilateral and bilateral abdominal retention (38/38 100%) and in 6/15 (40%) incomplete abdominal retention. An open inguinal orchiectomy was performed in all cases of inguinal retention (17/17, 100%) and in 9/15 cases of incomplete abdominal retention (60%). Unilateral cryptorchids having the other testis descended into the scrotum (*n*. 65) were castrated with an open standing approach (*n*. 40) [41] if undergoing laparoscopic cryptorchidectomy, and with an inguinal open approach with primary closure of the surgical wound (*n*. 25) if undergoing inguinal cryptorchidectomy in general anesthesia [45,46].

Information about the duration of sedation for standing procedures, of anesthesia for recumbent procedures, and of surgery were available for 49/70 patients (70%).

For standing laparoscopic cryptorchidectomy the mean duration of sedation was 68 ± 17 min (median 60 min, range 40–120 min), while the mean duration of the surgical procedure was 47 ± 13 min (median 45 min, range 30–65 min). Additional doses of butorphanol were required in 3/44 (6.8%) cases during laparoscopy, in one case during the abdominal insufflation with carbon dioxide, and in 2 cases at the first application of the Ligasure over the spermatic cord.

For open inguinal orchiectomy, the mean duration of anesthesia was 85 ± 22 min (median 90 min, range 45–130 min), while the mean duration of the surgical procedure was 59 ± 16 min (median 60 min, range 35–95 min). There was a statistical difference for total anesthesia and sedation time between the approaches used for surgery, as well as for the surgical time (Student *t*-test, *p* = 0.001). The cases of incomplete abdominal retention treated with open inguinal orchiectomy had a mean duration of anesthesia of 90 min (median 90, range 70–105 min), while the mean duration of surgery was 57 min (median 58.5, range 40–70 min).

Intraoperative complications during the laparoscopy were accidental spleen puncture during the surgical procedure (1/44, 2.2%), and moderate self-limiting bleeding from the spermatic cord in 10/44 cases (22.7%). Short-term complications were mild fever (38.5–38.8 °C) that developed in 3/44 cases (6.8%), and emphysema in 15/44 (34%), both resolving spontaneously without any adjunctive treatment. With regard to open inguinal orchiectomy, difficulties with identification of the testis in the inguinal canal during surgery were encountered in 8/26 cases (30.8%); in 6/8 of these cases (75%) the retention was incomplete abdominal. In these cases, an intraoperative gross inspection of the testes and their annexa was performed in order to ensure effective removal of all the structures. A moderate and self-limiting swelling of the inguinal region was observed in the majority of cases after surgery (17/26, 65.4%).

In cases of unilateral abdominal retention when a standing castration of the descended testis with secondary wound closure was performed, horses received a three-day antimicrobial therapy with penicillin-dihydrostreptomicyn (10,000 U.I. i.m. every 24 h). In all other cases, a preoperative broad spectrum antimicrobial therapy with ampicillin (20 mg/kg i.v.) and gentamicin (8 mg/kg i.v.) was guaranteed.

All horses received a three-day administration of NSAIDs (phenylbutazone 2.2 mg/kg i.v. every 24 h).

## 4. Discussion

In this case series the clinical records of horses referred for uni- or bilateral cryptorchidectomy between 2009 and 2019 were reviewed, analyzing breed prevalence, distribution of retained testicles, preoperative localization of the retained testis, surgical management and complications.

Concerning the breed predilection, in accordance with previous literature [38], we found that Western Riding horse breeds had the highest incidence of this disorder, although a retrospective study may have controversial results [7]. Despite Quarter horses being numerically superior (34/70), when we went through the medical records of the VTH, we observed a relatively high percentage of Appaloosa stallions, with regards to the population of horses referred to the VTH for the investigated disorder compared to literature (10/70, 14.3% vs. 38/604, 6.3%) [38], suggesting that this breed may potentially have a higher incidence than expected.

At our facility, diagnostic work-up for cryptorchidism is based on a comprehensive clinical examination of scrotal and deep inguinal palpation under sedation, a transabdominal percutaneous ultrasound, and palpation *per rectum*. Since the inclusion criteria of the study were the diagnosis of unilateral or bilateral cryptorchidism, and the surgical removal of the retained testes, localization of the retention was always achieved with the aid of one or more diagnostic tools. Palpation *per rectum* was performed in 30% of cases, when scrotal and inguinal palpation were not conclusive. However, this diagnostic approach has to be used cautiously and when strictly needed both because of its reliability, which is strictly associated with clinician experience [8,46,47], and because of the associated risks in young and fractious horses. Concerning the preoperative localization of the retained testis, we observed that transabdominal ultrasound was the most reliable method with 43.5% of positive diagnosis. Although our percentages were lower compared to those of Schambourg et al. (2006) [14] (43.5% vs. 97.8%) this technique appeared to be more reliable when compared with rectal and inguinal palpation. This difference can be ascribed to the experience of the clinician, the scanning method being more difficult than described [39].

As in previous published studies [8,48], we observed a majority of unilateral cryptorchid horses (65; 94.2%); particularly, left sided abdominally retained testes (28/65; 43%) as compared with right-sided ones, and of right inguinal retained testes (9/65; 14%) over left inguinal ones. Despite the low incidence of bilateral retention, we observed 7% of stallions with both retained testicles (5 cases out of 69), that were, in most such cases (3 out of 5), abdominal.

It is well known that for abdominally retained testicles, a standing laparoscopy offers many advantages over a recumbent laparoscopy in general anesthesia, and inguinal open cryptorchidectomy [8,12,26]. Since surgeons at our facility are familiar with standing laparoscopy, all abdominal testicles were removed with this technique. Although left flank unilateral approaches for bilateral abdominal retention have recently been described [49,50], a bilateral approach was used for bilateral abdominal retentions. Despite the bilateral abdominal approach, we could observe a shorter surgical time compared to that reported for a unilateral laparoscopy in bilateral abdominal retentions, probably because of better visualization and manipulation of the retained testis over the right flank with an ipsilateral flank access.

At the end of the standing laparoscopy, the removal of the excised abdominal testis was achieved through the widened ventral flank incision. Although intraoperative complications for this method are described (dropping of the testis into the abdomen, risk of parenchymal rupture) [27], none occurred. During the postoperative period, tissue dead space in the abdominal wall and trauma to abdominal muscles were responsible for moderate swelling and emphysema that occurred in a consistent number of cases (approximately one third) [51].

For the treatment of incomplete abdominal retention, a recumbent open inguinal access was chosen in the majority of cases (9/15), while standing laparoscopic cryptorchidectomy was selected in the remaining 6/15 cases. The choice of one technique over the other, relied mainly on the surgeon’s preference, although selection criteria should also include temperament and age of the horse. We observed that the most common surgical problem was the identification of the testis or its adnexa in the inguinal canal for incomplete abdominal retentions. Therefore, in these cases, prolonged surgical and anesthesia time were noticed.

Although laparoscopy can be challenging and even dangerous in juvenile or fractious horses, in older horses the risks associated with a standing open orchiectomy are higher, with a higher risk of herniation or eventration [12]; in such cases it might be preferred to remove the descended scrotal testis via an inguinal approach in dorsal recumbency with primary closure of the surgical wound.

In all cases, removal of the retained testis was achieved. Removing the testis is suggested to avoid possible revascularization of part of it, if excised but left in place, from anastomosing vessels derived from the cremaster or external pudendal artery [52] with persistence of stallion behavior.

The main intraoperative complication with the inguinal approach was the difficulty in identifying and grasping the structures associated with the testis (gubernaculum testis, epididymis) within the inguinal canal, once in dorsal recumbency.

In cases of unilateral inguinal cryptorchidism, removal of the scrotal testis was achieved via an inguinal open approach with primary wound closure [46], under general anesthesia, while in cases of abdominal cryptorchidism after the standing laparoscopic cryptorchidectomy, a standing scrotal orchiectomy with second intention healing [53] was performed. According to the literature, standing not sutured castration costs approximately one third as compared with recumbent sutured castration [54], but the complication rate is higher (39–46% vs. 2.7–8.5%) [55,56], the recovery periods longer, the postoperative management more demanding and sometimes cosmetic results worse [53,57]. In our cases, when presented with inguinal retention the choice of an open inguinal orchiectomy was straightforward. On the other hand, in cases of abdominal retention, a standing not sutured castration was preferred to reduce the duration of the procedure and avoid the risks associated with general anesthesia, which are considered more severe than those associated with a scrotal orchiectomy with second intention healing. In the recumbent horse, an inguinal approach to the descended testis was preferred over a scrotal approach for orchiectomy because fewer complications are described, probably due to lower susceptibility to surgical manipulation of the inguinal region compared to the scrotal one [46].

In the literature, the quantification and description of intra and postoperative complication rates are dependent on the chosen criteria, so the data are not always consistent and comparable. In this retrospective study a low rate of complications was reported, mainly intraoperative in horses with inguinal cryptorchidism, treated with open inguinal orchiectomy. No major postoperative complication such as hydrocele or funiculitis was observed, aside from a moderate and self-limiting swelling of the region during the post-operative period.

Although the evaluation of the sedative protocol was not the aim of the study, we nonetheless observed that the continuous infusion rate of detomidine associated with an initial bolus of butorphanol resulted in an adequate sedation for the procedure required, usually without the need for additional doses of opioids.

Seldom, during laparoscopic cryptorchidectomy, a self-limiting bleeding from the spermatic cord was observed. For the sealing of the testicular vessels together with the mesorchium, the Ligasure technology forceps ensured optimal results and avoided the need of intra and extracorporeal ligation of the cord, reducing the time required to complete the procedure [19,21,22,26,51]. According to the literature, this system provides hemostasis to vessels up to 7 mm in diameter [24]. Although we did not investigate the size of vessels of the mesorchium, we suppose that the bleeding could be related to an excessive thickness of tissues or to the attachment of necrotic tissue over the branches of the Ligasure forceps, hindering hemostatic power transmission.

In the majority of cases treated with a laparoscopic cryptorchidectomy, a mild subcutaneous emphysema was observed after surgery; in our study this condition was considered a primary postoperative complication, although other authors do not consider postoperative emphysema a real complication, but rather a variation on what is normal [16]. Actually, despite this, no horse showed any clinical sign of illness or discomfort. Therefore, no medical treatment was instituted other than parenteral flunixin, as for all other horses undergoing an orchiectomy.

Antimicrobial use in healthy patients undergoing elective surgery is considered controversial [38]. Although cryptorchid surgery is considered elective, a prophylactic use of a broad spectrum antimicrobial was chosen in order to avoid secondary infections that could be devastating [58]. Since antimicrobial therapy did not differ among patients, we could not evaluate the efficacy of one type over another one.

## 5. Conclusions

Although the retrospective nature of the study is its main limitation, as not all data were always available from the medical records, it confirmed that cryptorchidism is a widespread disease in the horse, affecting mainly the Western Riding horse breed population.

According to our results, the combination of inguinal palpation, transabdominal ultrasonography and transrectal palpation proved to be a reliable diagnostic procedure to detect the level of retention before planning surgery. For orchiectomy, a standing laparoscopy was confirmed as the preferred procedure for abdominal-retained testis with almost no complication. The inguinal testes were removed safely in general anesthesia and dorsal recumbency with a standard inguinal approach. The incomplete abdominal-retained testicles were better removed in a standing laparoscopy.

The inguinal recumbent approach was a viable option for concurrent orchiectomy of a contralateral descended testis, although the procedure was often prolonged.

However, these assessments need to be confirmed in a larger study involving more equine practices in order to have more consistent data.

## Figures and Tables

**Table 1:** Values are reported as number (%).

Location	Left (*n* = 44)	Right (*n* = 21)	Bilateral (*n* = 5)
Abdominal	28 (64)	7 (33)	3 (60%)
Incomplete abdominal	9 (20)	5 (24)	1 (20%)
Inguinal	7(16)	9 (43)	1 (20%)
